# How useful is Nanopore adaptive sampling for sequencing *Schistosoma mansoni* miracidia?

**DOI:** 10.12688/wellcomeopenres.24094.1

**Published:** 2025-05-16

**Authors:** Rivka M. Lim, Thomas M. Arme, Moses Arinaitwe, Andrina Barungi, Thomas Crellen, Arne Jacobs, Amy B. Pedersen, Joanne P. Webster, Poppy H.L. Lamberton

**Affiliations:** 1 The University of Edinburgh Institute of Evolutionary Biology, Edinburgh, Scotland, UK; 2 University of Glasgow Institute of Biodiversity Animal Health and Comparative Medicine, Glasgow, Scotland, UK; 3 Vector Borne and Neglected Tropical Diseases Control Division, Ministry of Health, Kampala, Uganda; 4 The Royal Veterinary College Department of Pathobiology and Population Sciences, Hatfield, England, UK

**Keywords:** Nanopore, Adaptive sampling, Schistosomiasis, Miracidia

## Abstract

**Background:**

Whole-genome sequencing (WGS) is now widely used in *Schistosoma* genomics. Whilst adult worms typically provide sufficient DNA for molecular analyses, their inaccessibility in live definitive hosts presents a challenge for population studies. Larval stages, such as miracidia can be collected non-invasively and preserved on Whatman FTA cards, however these samples typically yield low quantities of DNA and have high levels of contamination, particularly when obtained from stool samples. To counteract contamination, multiple washing steps prior to placement onto Whatman FTA cards can be performed, but this is labour-intensive and can limit the number of larvae collected.

**Methods:**

Nanopore sequencing technologies includes an “adaptive sampling” feature, which enables selective enrichment or depletion of target DNA sequences during sequencing. In this study, we evaluated the potential of adaptive sampling to selectively enrich *S. mansoni* DNA from both washed and unwashed larval stage miracidia. We used Kraken2 to characterise sample contamination and assessed sequencing breadth and depth of genome coverage to determine whether adaptive sampling could provide sufficient *S. mansoni* DNA for WGS.

**Results and conclusion:**

Our results demonstrate that washed samples contained a higher proportion of *S. mansoni* DNA, validating the effectiveness of washing for contamination removal. However, adaptive sampling failed to generate sufficient *S. mansoni* reads for effective WGS. These findings suggest that, at present, washing remains critical for maximising *S. mansoni* DNA purity as adaptive sampling alone is insufficient for enrichment. Alternative enrichment strategies will be necessary to improve sequencing efficiency and data quality for *S. mansoni* WGS.

## Introduction

Schistosomiasis is a disease prevalent in tropical and subtropical regions which, despite decades of control programs remains a significant public health challenge in many countries ^1^. It is caused by parasitic trematodes of the genus *Schistosoma* and is responsible for considerable morbidity and socioeconomical burden in affected communities. A person or animal who is infected with *Schistosoma* will excrete parasite eggs into the environment with faeces or urine (depending on the species). If an egg contacts fresh water it will hatch into a miracidium, which can infect specific species of freshwater snails ^2^. Within this intermediate host the parasite undergoes asexual reproduction until thousands of clonal copies are released back into the water as cercaria ^3^. This is a free-living motile stage which can then swim to find a mammal definitive host, penetrate the skin, travel through the body to the liver, mature into adult worms and then travel to the venules surrounding either the bladder or the intestines ^4^. Here male and female pairs can survive for decades and are completely inaccessible, whilst the host is alive ^5^.

To inform control strategies and carry out disease surveillance, including monitoring potential drug resistance and the development of effective vaccines, an understanding of the genetic diversity and population structure of parasites is essential ^6– 8^. While targeted sequencing approaches, such as microsatellite genotyping and amplicon-based analyses, remain invaluable for large-scale population studies where high-throughput screening of specific loci is necessary, whole-genome sequencing (WGS) provides comprehensive insights into parasite genomes ^9– 11^. Previous studies using microsatellite panels ^12^, revealed extensive panmictic gene flow of *Schistosoma* across Africa. This high genetic diversity makes it challenging to detect population structure at broad scales, WGS can be used to uncover micro-heterogeneity and evidence of selection pressures. This more zoomed-in approach makes WGS particularly well-suited for identifying subtle population structures and genetic changes. Furthermore, advances in sequencing technologies have significantly reduced the costs associated with WGS, making it an increasingly accessible option for parasite research ^13^.

The inability to directly sample adult *Schistosoma* worms due to their location in venules deep within the body poses significant challenges for molecular epidemiological studies of these parasites. While some insights have been gained from human autopsy data ^14^, these are not representative of endemic populations. To address this limitation, laboratory models employing indirect sampling methods have been used to generate adult worms. These models involve infecting laboratory snails with miracidia to produce cercariae, which are then used to infect laboratory mammals ^15^. However, studies have demonstrated that laboratory passage not only reduces the genetic diversity observed in wild populations, but also imposes selection pressures specific to the choice of laboratory host used for passage, rendering these models unrepresentative of the molecular diversity found in natural settings ^16, 17^. Although much of the recent focus has been on *Schistosoma* spp., similar challenges in obtaining adult-stage material for molecular analyses are shared across other helminths of public health importance. In these cases, larval stages, which are more readily accessible in the environment or from host excreta, offer a practical alternative for DNA sampling. These are often preserved on Whatman FTA cards; however, such samples can yield limited DNA and are prone to contamination, especially when sourced from stool. Therefore, the most biologically representative, cost-effective and least invasive technique for genotyping *Schistosoma* from natural infections in definitive hosts involves the use of miracidia ^18^, providing access to genetic information about the parental worms. To isolate individual miracidium from intestinal parasites, faecal samples are washed, filtered and the resulting sediment incubated in fresh water under light conditions to stimulate hatching, followed by manual collection of each individual miracidium using a light microscope and a pipette ^18^. Miracidia are typically preserved on Whatman Indicating FTA cards (hereby called FTA cards). These cards are covered in a coloured dye which is cleared upon contact with liquid, therefore it is possible to store up to 1000 individual miracidia on one card and easily identify the location of the parasite in the dye-cleared area. Enzymes within the card lyse the cells, digest the proteins and bind the DNA, enabling the long-term stabilisation of nucleic acids at ambient temperatures, making them ideal for field studies in remote or resource-limited settings. By preserving individual miracidia on FTA cards, researchers can transport thousands of miracidia to laboratories for DNA extraction and subsequent genetic analysis. The Schistosomiasis Collection at the Natural History Museum (SCAN), for example, has demonstrated the value of this approach, archiving miracidia samples from across endemic regions collected by many independent researchers for over a decade and distributing them to scientists around the world for further research ^19^.

Despite the advantages of preserving individual miracidia, there are still some issues with this technique particularity for WGS. The parasite has a relatively large genome, with the three main *Schistosoma* species that infect humans having 363 – 409 mega bases ^20– 22^. Furthermore, the miracidium is the smallest stage of the life cycle with approximately 365 cells in contrast to tens of thousands of cells in an adult worm ^23^, therefore amplification is required to obtain sufficient DNA for either targeted or whole genome sequencing (WGS). Another major challenge when using miracidia for WGS is contamination, since DNA from host cells, bacteria, and other faecal contaminants often co-extract with *Schistosoma* DNA and are then amplified along with the *Schistosoma* DNA complicating downstream genomic analyses ^24^. To combat this, miracidia are washed multiple times in clean water before preservation on the FTA cards to minimise environmental DNA contamination. Whilst this method improves sample purity, it is extremely labour and time-intensive and therefore significantly limits the number of miracidia that can be stored in a given time. This becomes further exacerbated in samples from people with lower infection intensities as miracidia can be lost during the washing process.

The most promising development thus far for enrichment of parasite DNA in contaminated samples has been with exome capturing, which isolates target exome DNA sequences using hybridisation to RNA baits ^25^. This technique has been used to successfully isolate *S. mansoni* DNA from archived miracidia preserved on FTA cards at a high coverage (96% on average) and depth (52x average) ^24^ and due to the targeted nature removes most contaminants. However, this technique has the disadvantages of not sequencing non-coding regions and also, due to the custom-designed probes, not capturing highly variable genes which have significant sequence divergence. In parasite population-level studies, genes which are highly variable (i.e., those with many different alleles or mutations across individuals and populations) are particularly informative for examining genetic diversity, adaptation, and evolution. Poor capture efficiency for these genes could thereby reduce the ability to detect key variants. Therefore, a technique which could be used directly on miracidia without repeated washing would enable cost-effective streamlining of future studies allowing storage from more people, increasing the likelihood of having samples from people of interest. Also, this new method would enable the retrospective analysis of unwashed, archived samples, such as those stored at SCAN. These samples could then be processed using WGS, with the added advantage of *a priori* knowledge of which samples are most critical for sequencing.

Adaptive sampling, a unique feature of Nanopore Technologies, is a method of software-controlled enrichment which enables selective enrichment of DNA regions or organisms in real-time sequencing ^26^. During adaptive sampling, a DNA fragment begins to pass through the nanopore on the flow cell and the initial sequence is rapidly aligned to a reference sequence or genome to approximately 400 bases. Based on this alignment, the read is either accepted, where sequencing continues to the end of the DNA fragment, or rejected in which the voltage across the nanopore is reversed and the pore is free again to sequence a different section of DNA. By dynamically rejecting unwanted reads and only sequencing reads which align with a reference sequence, adaptive sampling has the potential to maximise the yield of target DNA and deplete contaminant DNA. One study which used adaptive sampling to enrich for *Plasmodium falciparum* in blood samples, observed a 3–5-fold increase in DNA yield in comparison to regular Nanopore sequencing ^27^. This approach therefore has the potential to overcome many limitations associated with miracidia isolation and contamination, offering a scalable and efficient method for generating whole-genome data directly from FTA card-stored samples, and potentially and most critically, including unwashed samples.

In this study, we focused on *S. mansoni*, the species responsible for most intestinal forms of schistosomiasis in humans in Africa ^1^. We assessed whether the adaptive sampling feature of Nanopore Technologies could be used to selectively enrich *S. mansoni* DNA, thus removing the contamination from samples which had undergone different processes prior to preservation on FTA cards. Whilst all stool samples were flushed with water, the individual miracidia were either preserved without washing (unwashed), or washed three times before preservation (washed). First, we characterised the relative effectiveness of washing prior to storage in reducing non- *S. mansoni* DNA contamination in comparison to the unwashed group. Second, to validate the results from the adaptive sampling, we compared proportions of *S. mansoni* DNA detected by this method with those identified through the k-mer based taxonomy tool Kraken2, which incorporates a comprehensive genomic database ^28^. This comparison also enabled us to characterise the contaminants present across all samples (i.e. the DNA that was not sequenced to the end of the fragment, but only for approximately 400 bases until the process rejected the fragment). Third, we evaluated the effectiveness of adaptive sampling in the unwashed group for WGS. Specifically, we estimated the coverage and depth of enriched *S. mansoni* sequences when mapped to the *S. mansoni* reference genome to determine whether this method can achieve the necessary quality for comprehensive genomic analysis.

## Methods

### Ethics statement

Ethical approval was obtained from the Ugandan Ministry of Health Vector Control Division Research Ethics Committee (reference: VCDREC/062, 24/08/2021, extension 06/05/2022), the Uganda National Council for Science and Technology Social Sciences (reference: UNCSTHS 2193, 24/08/2021, extension 06/05/2022), and the University of Glasgow College of Medical, Veterinary, and Life Sciences (reference: MVLS: 200160068,07/02/2017, extension 22/01/2019). Prior to any data collection, informed written consent was gained from all participants aged 18 years and above, either through a signature or thumbprint. For participants under the age of 18, consent was provided by parents or legal guardians also through a signature or thumbprint, and informed verbal assent was obtained from children aged 8 to 18 years. Participants were fully informed of their right to withdraw from the study at any time without any impact on their access to healthcare, including any treatment provided by the study or the national control programme. At the conclusion of the study, all participants received a snack and were offered praziquantel (40 mg/kg) and albendazole (400 mg). Those diagnosed with malaria were provided with dihydroartemisinin/piperaquine, which was administered directly to participants or, in the case of minors, to their parents or guardians.

### Sample collection

Sample collection was performed as part of a larger study which took place in Bugoto Village, Mayuge District, Uganda in May 2022, described in more detail elsewhere ^29^. Three stool samples were collected from each participant over three separate days and duplicate Kato-Katz thick smears processed and read under x100 magnification (CX21100 Olympus, Ste A Hicksville, NY 1180, United States) for visualisation of *S. mansoni* eggs ^30^. For the current study we used a single stool sample from one participant, a 7-year-old male and isolated multiple miracidia from this single sample. Malaria diagnosis was carried out by using finger prick blood from each participant and a rapid diagnostic test (RDT) (Ag P.f/Pan Malaria Rapid Diagnostic test, Standard Diagnostics Inc.) ^31^.

### Washing miracidia

Approximately 2 grams of faecal matter was homogenised with ~50 ml locally available, non-chlorinated bottled water before being pushed through a 200 µm metal sieve using a toothbrush, with approximately 1 L of bottled water. The recovered contents were then transferred to a Pitchford funnel which has two differing sized meshes, the first of which is 200 µm in size to allow the eggs to pass through but retain the larger faecal debris, the smaller mesh is 40–50 µm and retains the eggs ^32^, and both funnels washed using an additional 1L of bottle water. The sediment in the smaller mesh was then washed again until the solution looked clear, and the contents were collected in an opaque pot and stored overnight in the cool and dark. The following day the contents were collected in a clean glass petri dish and placed in dappled sunlight or under a bright lamp to induce the hatching of the miracidia from the washed eggs.

In the case of the unwashed samples, individual miracidia were then isolated directly from the water using a binocular microscope and a pipette, picked up in approximately 3 µl of water and spotted onto an FTA card. In the case of the washed group, the isolated miracidia were individually picked up and transferred to a petri dish of clean bottled water, with up to 30 miracidia placed into the clean water in total. This was then repeated twice more in fresh, clean bottled water before individually storing them on the FTA card. The cards were allowed to air dry for 1 hour and then stored for transport in sealed Ziplock bags with desiccants until DNA extraction.

### Whole genome amplification

Samples were prepared for whole genome amplification (WGA) following methods in Le Clec’h *et al.* ^24^. Briefly, a disc of FTA card was punched out using the Harris 2mm hole punch, from dye cleared regions which should only include a single miracidium. The 2 mm disc was then washed 3 times using 200 µl of FTA wash buffer for 5 mins whilst rotating. The FTA buffer was removed from the discs by rinsing using 200 µl TE ^-1^ buffer two times and dried at 56°C for approx. 10 mins.

WGA was carried out using GenomiPhi V2 DNA amplification kit. 9 µl of sample buffer was added to the FTA disc and a positive and negative control added to the experiment using kit instructions. The DNA on the FTA card matrix was denatured for 3 mins at 95°C and then 9 µl of reaction buffer and 1 µl of Phi29 polymerase added to each tube containing the FTA disc and sample buffer. Genomes were amplified for 2hrs 30 mins at 30°C and then 10 mins at 65°C. Once complete the samples were eluted in 100 µl of sterile water to have enough library to run twice with flow cell washing in between. The concentration, purity and fragment length were estimated for each sample using Qubit High-Sensitivity DNA Kit (Invitrogen), nanodrop and TapeStation system (Agilent) respectively.

The samples were cleaned using AMPure beads at a 1.4x bead to sample ratio, following the manufacturer’s instructions.

### Library preparation and sequencing

DNA repair and end preparation was carried out using the ligation kit V14 (SQK-LSK114) and New England Biolabs companion module v2 (E7672S) following the manufacturer’s instructions and 1 µg of DNA. Post library preparation and pre sequencing, the fragment length of samples was reassessed using the Tape Station. The peak fragment length was used as an average length to enable estimation of molecular concentration. Each sample was aliquoted into at least two libraries with a concentration as close to 50 fmol per library as possible.

Prepared libraries were sequenced on the MinION Mk1B, using FLO-MIN114 flowcells. To enable high data acquisition from each sample, one sample was run on each flow cell with no barcoding. MinKNOW version 24.02.6 was used for sequencing, adaptive sampling option was chosen and the Version 10 *S. mansoni* reference genome added for enrichment ^33, 34^. Live basecalling was disabled and one POD5 data file was generated each hour. A known issue during adaptive sampling is that pores become blocked relatively quickly and cannot accept any new DNA fragments (Nanopore Technologies, 2024), therefore sequencing was paused when all available pores were approximately 50% saturated, the flow cell was washed using the Flow Cell Wash Kit XL (EXP-WSH004-XL) and the second library added and sequencing resumed, this was repeated for all available libraries for each sample.

### Post sequencing analysis

Basecalling was carried out using Dorado (version 0.8.1) and the super accurate model ^35^. Using the adaptive sampling report, which is generated during sequencing, any read which had “stop_receiving” in the decision column was deemed to have passed the enrichment. As adaptive sampling uses its own independent basecalling mode, not all these reads were also available in the basecalled FASTQ files obtained using Dorado, therefore the FASTQ files were searched for matching read ids and all those which had been identified from the adaptive sampling file were marked as enriched and the percent of the total number of reads for each sample used in the analysis. A Shapiro-Wilk test for normality was carried out for the distribution of washed and unwashed groups. If both groups were normally distributed, a two-sample t test was performed and if not normally distributed a Mann-Whitney U test was performed to test for differences in the proportion of *S. mansoni* DNA for each group.

During adaptive sampling a DNA fragment is either accepted or rejected. However, the data from the partially read rejected fragments, although truncated to around 400–1000 basepairs, are saved in the output folder so are available for analysis. A custom Kraken2 ^28^ database was created using all reference bacterial, viral and protozoa genomes available on the NCBI refseq database { https://www.ncbi.nlm.nih.gov/datasets/}. Plus, the human reference genome, the UNI_vec_core database and finally the V10 *S. mansoni* reference genome. Kraken2 was ran using a 0.1 confidence cut off ^36^.

Kraken2 estimated a higher number of *S. mansoni* DNA sequences compared to those identified during the adaptive sampling process. To explore this discrepancy, sequences identified as *S. mansoni* by Kraken2, but not during adaptive sampling, were extracted and saved into individual FASTA files for each sample using seqtk (version 1.4). A custom BLAST database was created using all *Schistosoma* sequences available on NCBI under taxonomy code 6181. Each FASTA file containing sequences identified by Kraken2, but not adaptive sampling, was then subjected to BLAST (version blast+/2.9.0) searches against this database. Given the large number of sequences in some samples (tens of thousands), only the top hit for each query was reported. A data frame was generated summarising the results, including the total number of sequences aligning with the *Schistosoma* database and the counts for each species identified. The sequences which were a positive hit on BLAST were then realigned to the *S. mansoni* reference genome using MiniMap2 (version 2.28) ^37^.

To evaluate if the adaptive sampling process generated sufficient data for WGS, Nanofilt (version 2.8.0) ^38^ was used to trim the enriched reads to >150 bps and quality score >12 (mean quality score of all reads) and then they were mapped to the reference genome using MiniMap2 (version 2.28) ^37^. The total number of mapped bases and reads were obtained from the Samtools (version 1.21) stats and flagstats reports respectively ^39^. The number of times a base position was covered (depth of coverage) was obtained using Samtools (version 1.21) and the proportion of the reference genome covered by at least one read that mapped uniquely was used to assess breadth of coverage. Mean depth of coverage, was defined as the mean number of reads aligning to individual positions across the reference genome. Depth and breadth of coverage were further analysed using R (version 4.4.2), when plotting graphs, bins of 25kb were used for nuclear genomes and W or Z sex chromosomes and 2kb for the mitochondria.

## Results

### Washed miracidia have a higher proportion of *S. mansoni* DNA than unwashed miracidia

**Figure 1.**
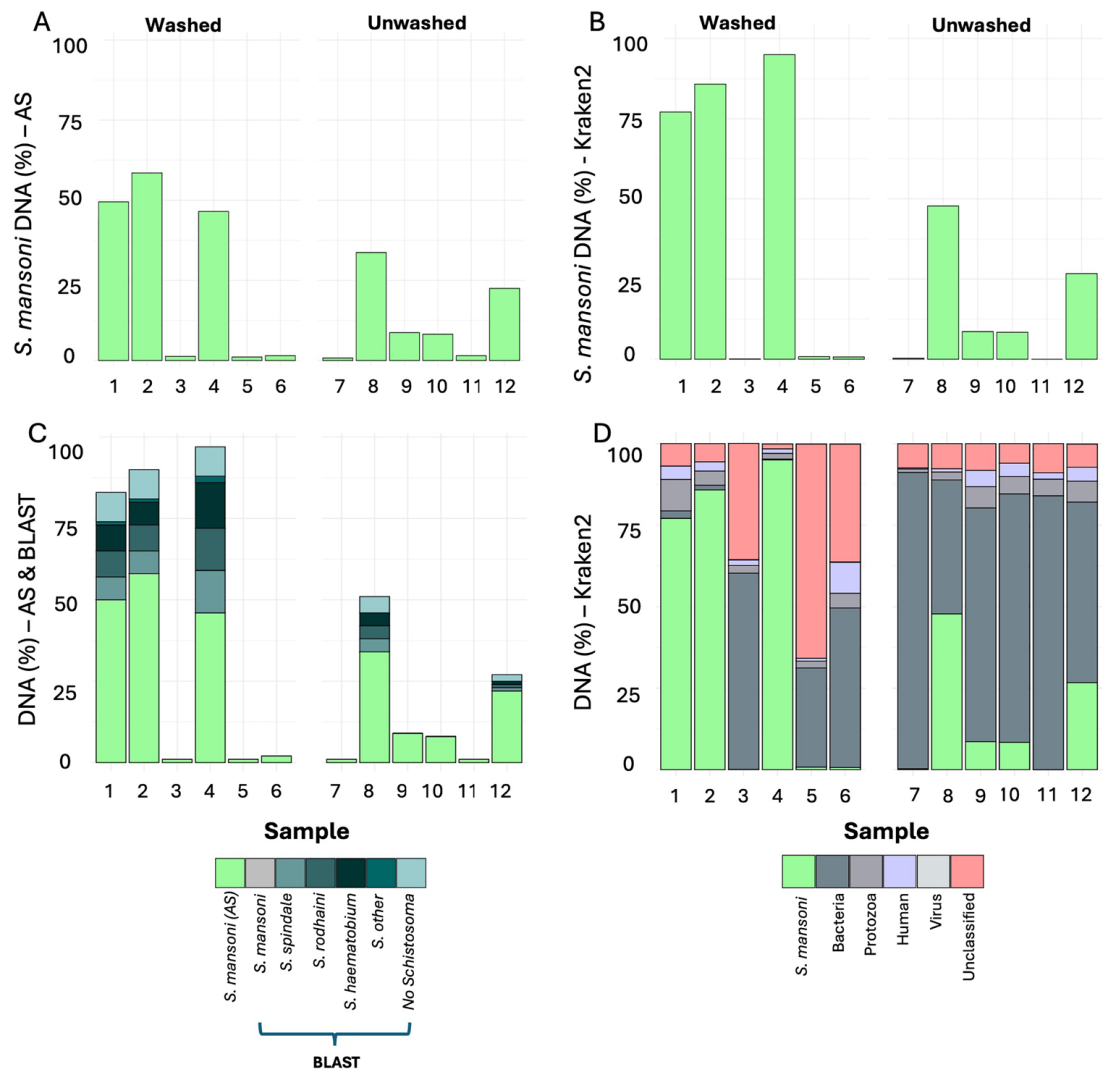
Proportion of *Schistosoma mansoni* DNA in samples using adaptive sampling and Kraken2. Twelve miracidia samples extracted from Whatman Indicating FTA cards, six washed pre-storage on the card and six unwashed pre-storage, were sequenced using Nanopore technology. Here we show the proportion of each sample that was identified as *S. mansoni* DNA when using Nanopore adaptive sampling [AS] ( **A**), and a Kraken2 custom database which included bacterial, viral, protozoa, human and *S. mansoni* reference genomes ( **B**). The sequences which Kraken2 identified as *S. mansoni* and AS did not were BLAST searched against a S *chistosoma* database ( **C**). Contamination was estimated using Kraken2 ( **D**).

Samples containing washed miracidia had a higher proportion of *S. mansoni* DNA within the whole DNA pool than the unwashed group, with 27% and 13% of reads mapping respectively, but this difference was not statistically significant (W = 21.5, p= 0.630). However, five out of the 12 samples had below 1.6% (mean= 1.2%) *Schistosoma* DNA enriched during the adaptive sampling process (samples 3, 5, 6 in washed group and 7, 11 in unwashed group) ( Figure 1A), indicating that either the adaptive sampling process failed or there was not a miracidium in the sample. When these samples were removed the overall percent of *Schistosoma* DNA increased to 53% in the washed group and 18% in the unwashed group and the difference between the two groups was statistically significant (t=4.24, df=5, p=0.008).

### Kraken2 estimates a higher proportion of *S. mansoni* DNA compared to adaptive sampling

When using the custom Kraken2 database which included the *S. mansoni* reference genome version 10 ^33, 34^, the overall amount of *S. mansoni* DNA estimated in each sample was higher than the adaptive sampling. This result was particularly pronounced in the washed group in comparison to the unwashed group with 43% and 15% respectively ( Figure 1B). Similar to the adaptive sampling results, the difference between the two groups within Kraken2 analysis was not statistically significant (W=23, p=0.484). The five failed samples, which had <1.6% DNA in the adaptive sampling, also had very low *S. mansoni* DNA concentration in the Kraken2 evaluation (<0.8% and average 0.4%). Once these failed samples were removed from the analyses there was a statistically significant difference between washed (86%) and unwashed (23%) groups, (t=5.3, df =5, p=0.003). When comparing the amount of DNA in the washed and unwashed groups between adaptive sampling and Kraken2 (with the five samples removed), there was no statistical difference between the unwashed groups (t = 0.4, df = 6, p= 0.694) but there was a statistical difference between the washed groups, with the Kraken2 estimation of *S. mansoni* statistically higher than in the adaptive sampling group (t = 5.5, df = 4, p=0.005).

To determine why Kraken2 identified a statistically higher amount of *S. mansoni* DNA in the washed group in comparison to adaptive sampling, the sequences that Kraken2 identified as *S. mansoni*, but adaptive sampling did not, were BLAST searched against a *Schistosoma* database. This confirmed that many of these sequences were most similar to alternative species of *Schistosoma* parasite, with the top three being *S. haematobium*, *S. spindale* and *S. rodhaini*, with the mean of all BLAST results for each sample being 37%, 32% and 28% respectively ( Figure 1C). The sequences that had been rejected by adaptive sampling but were positive for *Schistosoma* during the Kraken2 analysis and confirmed as *Schistosoma* by BLAST were then mapped back to the reference *S. mansoni* genome, 90% were mapped successfully to the genome, but only 12% of these were primary mapped, with a very high number of secondary mapped alignments which are reads which could be aligned in multiple regions.

Kraken2 confirmed the majority of the contamination in these samples was bacteria, with a small proportion of human and protozoan DNA. On further investigation the latter finding was predominantly *Plasmodium* DNA indicating that this participant was also infected with malaria at the time of collecting the faeces ( Figure 1D) which was also confirmed by a positive RDT test for *Plasmodium*. As the samples with low *S. mansoni* DNA had a high number of bacteria this indicates that the sequencing was likely successful but there were no miracidia present in the failed sample. However, samples 3, 5 and 6 had a relatively high amount of the sequences unclassified by Kraken2 which suggests that some of the contamination here was taxa not in the database or a high number of sequences were unreadable which could be due to sequencing error or sample quality.

### Adaptive sampling with nanopore sequencing is not an effective method for enriching sufficient DNA for WGS from miracidia samples

**Figure 2.**
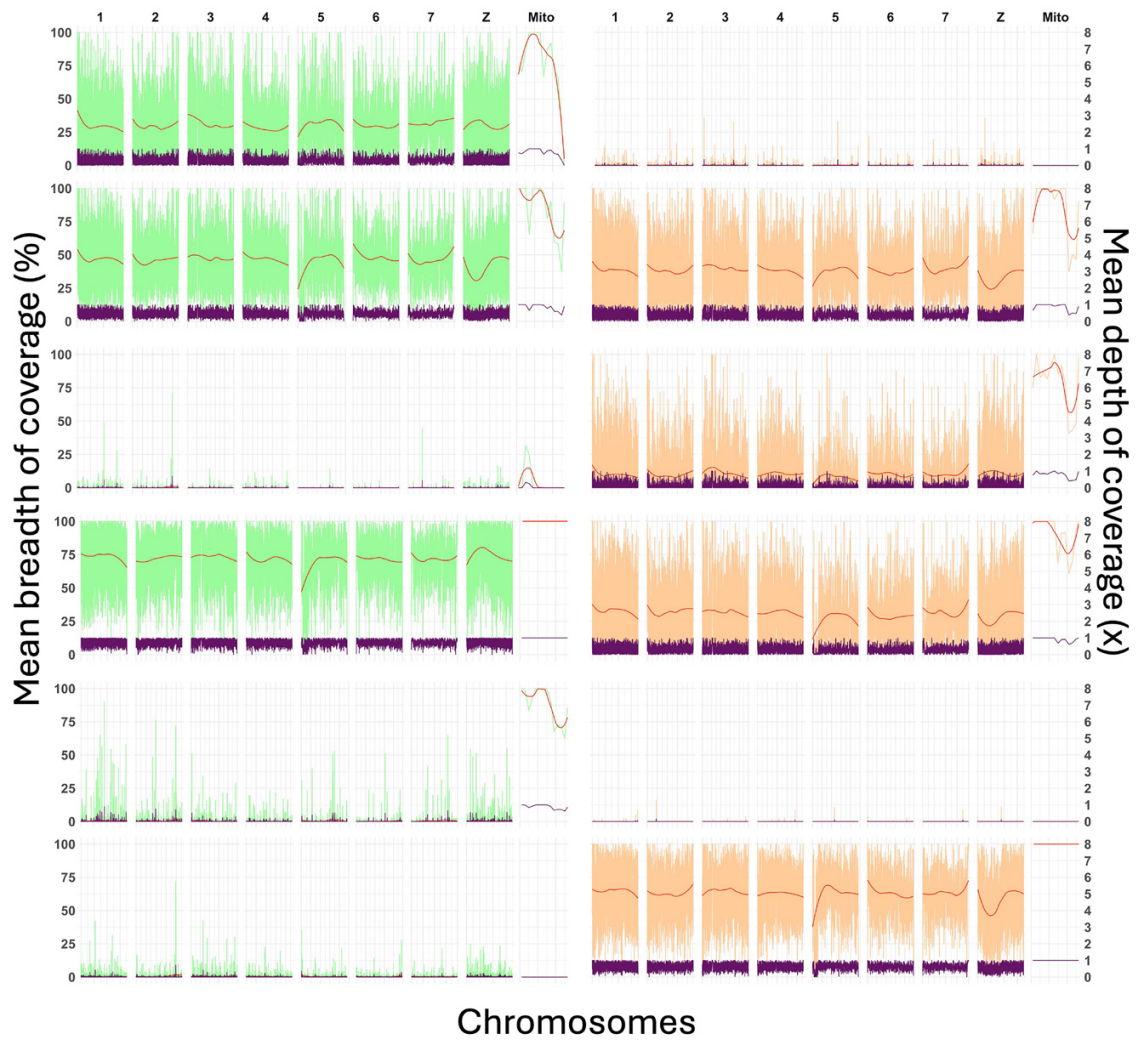
Mean breadth and depth of coverage when mapping enriched sequences to the *Schistosoma mansoni* reference genome (V10). Twelve individual miracidia samples extracted from FTA cards, either washed (green [1–6 vertically]) or unwashed (orange [7–12 vertically]) were sequenced and mapped to the *S. mansoni* reference genome (V10). Mean breadth of coverage per 25kb bin is plotted for each nuclear chromosome and 2kb bins for mitochondrial chromosome on the y axis (green or orange), the smoothed mean is indicated in red. The secondary y axis indicates the depth of coverage and is in purple.

Out of the 12 samples which were tested in this study, depth and breadth of coverage were very low and varied greatly. The mean breadth of coverage of the nuclear genome ranged from 0.01% to 67% and depth ranged from 0.00 to 2.5x ( Table 1 and Figure 2). There was an average coverage of 22% in the washed samples and 21% in the unwashed samples. Only three samples (2, 4, 12) had a depth of >1x but as mentioned previously these did not have a high number of bases covered, with 40%, 67% and 57% respectively ( Table 1 and Figure 2).

**Table 1.** Depth and breadth of coverage statistics of adaptive sampling enriched reads against *Schistosoma mansoni* reference genome.

		Nuclear		Sex		Mitochondrial	
	Sample	Mean Breadth (%)	Mean Depth (x)	Mean Breadth (%)	Mean Depth (x)	Mean Breadth (%)	Mean Depth (x)
Washed	1	25.63	0.63	1.43	0.03	54.58	2.60
	2	40.55	1.07	22.15	0.58	66.95	2.35
	3	0.07	0.02	0.00	0.02	4.19	0.09
	4	67.25	2.54	3.12	0.13	92.87	14.55
	5	0.32	0.12	0.04	0.05	71.70	5.00
	6	0.30	0.01	0.03	0.01	0.00	0.00
Unwashed	7	0.08	0.01	0.02	0.35	0.00	0.00
	8	32.60	0.68	16.25	0.51	61.16	5.13
	9	7.92	0.18	0.62	0.02	57.71	1.54
	10	25.96	0.52	7.20	0.27	66.52	5.10
	11	0.01	0.00	0.02	0.13	0.00	0.00
	12	57.35	1.60	23.93	0.70	87.34	14.50

Mitochondrial breadth of coverage was on average higher with 47% in washed and 45% in unwashed and ranged from 0-14.5x depth. Breadth of coverage of the sex chromosome was generally very low with a max of 22% in the washed samples and 24% in the unwashed samples ( Table 1). Mean map quality scores were on average high (30–56 for the nuclear genome), indicating that most reads align uniquely and accurately to the reference genome.

## Discussion

Nanopore adaptive sampling could not generate a sufficient quantity of DNA to enable effective WGS of *S. mansoni* from single miracidia preserved on FTA cards. Therefore, in this study adaptive sampling was not a viable method for eliminating contamination and circumventing the need for labour-intensive washing of miracidia in the field setting prior to storage.

In addition, there was a low success rate for isolating miracidia in the field, with 5 out of the 12 samples not having miracidia DNA amplified at all, indicating that a miracidium was possibly not present. This is in line with previous findings from 191 *S. mansoni* samples preserved on FTA cards, where only 51% contained *S. mansoni* DNA post whole genome amplification ^24^. When removing these failed samples from the analysis, washed samples contained a significantly higher proportion of *S. mansoni* DNA than the unwashed ones which validates the technique of washing to remove some contamination for WGS.

Our analysis using Kraken2 identified a statistically higher proportion of *S. mansoni* reads in comparison to the adaptive sampling process. Given previous evidence of Kraken2 overestimating taxa especially in low confidence cutoffs (0.1) as used in this study (Liu *et al.*, 2024), and so to test whether this was a true result, we then BLAST searched all the sequences identified as *S. mansoni* by Kraken2, but not identified by adaptive sampling, and found that the majority were positively identified to the *Schistosoma* genus with top hits for *S. haematobium*, *S. rodhaini* and *S. spindale*. Species of *S. spindale* are found across South-East Asia ^40^, and although *S. haematobium* may have been found in this region in the past, this species is unlikely to be able to produce viable hybridizations with *S. mansoni* as the species are so distantly related ^41^, therefore genomic introgression from historic or recent hybridisation is extremely unlikely here ^42^. Therefore, it is likely that these reads do originate from *S. mansoni*, despite the failure of adaptive sampling to enrich them. Indeed, when these reads identified as other species were mapped back to the *S. mansoni* reference genome, 90% aligned successfully, strongly suggesting their parasite origin is *S. mansoni*. However, the high frequency of secondary alignments observed suggests that repetitive genomic regions may have impeded the efficiency of the adaptive sampling process. Nanopore adaptive sampling uses the primary alignments to make decisions about whether to retain or reject reads, secondary alignments are not considered in this process (personal communication with Oxford Nanopore Support team). For organisms with large and complex genomes, such as *S. mansoni* (363 Mb), this poses a challenge. The *S. mansoni* genome contains many repetitive regions, comprising approximately 40–50% of the genome ^20^, these repeats result in a high frequency of secondary alignments because reads originating from repetitive regions can match multiple locations in the genome with similar alignment scores. Because adaptive sampling only evaluates primary alignments, it may inadvertently reject reads that belong to *S. mansoni* if they fail to align uniquely. Our analysis of rejected DNA reads provides some evidence to support this hypothesis, however, because the rejected reads are truncated at approximately 400 base pairs, it is challenging to definitively determine the extent to which repetitive regions or alignment ambiguity contributed to the issue. Nonetheless, these findings highlight a key limitation of adaptive sampling for *S. mansoni*, where the genome complexity could hinder enrichment.

To better characterise the rejected reads, a comparative experiment dividing the flow cell between adaptive sampling and standard sequencing modes would be valuable, as this would allow for the requisition of full-length versions of the reads which were rejected in adaptive sampling. This was not carried out in the present study due to the fact that it would have further reduced the overall yield, which was already a limitation of the study outputs. This low yield also facilitated a further limitation of the study, the low sample size, with only six miracidia sequenced per group. The low DNA yield from miracidia necessitated singleplexing during the sequencing to maximize data recovery from each sample during test runs. However, singleplexing is prohibitively expensive, with an average cost of £700 per sample which includes flow cells and library preparation reagents. Once it became apparent that the breadth and depth of coverage achieved would be insufficient for downstream analysis, additional sequencing runs were not pursued as were not economically viable for the output being achieved. For future studies, the design of a multiplexing strategy would be essential to improve cost-effectiveness.

An outcome of note from the Kraken2 analysis was being able to characterise the contamination across samples. As could have been predicted in samples which are derived from faeces, the majority of contamination was from bacteria. Furthermore, it was possible to detect that the participant was positive for malaria with an average of 4% of the reads being identified in the *Plasmodium* genus.

Mapping the enriched reads to the reference genome revealed insufficient DNA to achieve full genome breadth of coverage or meaningful depth. A known challenge in sequencing *Schistosoma* miracidia is the low DNA yield, as each miracidium consists of only approximately 365 cells ^23^ and it is not feasible to recover all these cells during the extraction process ^24^. The three washed samples which definitely contained miracidia demonstrated the highest breadth of coverage 26%, 41% and 67%, and depth 0.6x, 1.07x and 2.5x respectively. It is not possible to estimate the potential coverage if all the *S. mansoni* DNA identified by Kraken2 had been mapped to the genome. Using multiple reference genomes which encompass all *Schistosoma* species, or a generalised *Schistosoma* reference genome, could theoretically enhance DNA enrichment across samples. However, we do not believe that this is possible with the current MinKNOW software. Nevertheless, the main objective here was to evaluate the feasibility of using unwashed samples and there was no significant difference in the number of *S. mansoni* DNA reads between adaptive sampling and Kraken2 in the unwashed samples. This indicates that a high proportion of available *S. mansoni* DNA was enriched, but the total DNA yield was insufficient to achieve WGS. A key issue with unwashed samples is the extremely high levels of contamination, resulting in amplification of both target and non-target DNA prior to sequencing. In these unwashed samples, the majority of reads consisted of contaminant DNA, leading to rejection during sequencing. Consequently, only a small proportion of reads were available for mapping to the *S. mansoni* genome, severely limiting the overall sequencing efficiency and depth.

The primary difference between the groups analysed in this study was the number of washes performed (three for the washed group and zero for the unwashed), and it would be valuable to determine whether a single or double wash could yield comparable results. This modification could reduce contamination and reduce labour intensity.

## Concluding remarks

This study demonstrates that Nanopore adaptive sampling, in its current form, is not a viable method for selectively enriching *S. mansoni* DNA from unwashed miracidia on FTA cards. The low DNA yield from individual miracidia, combined with the limitations of adaptive sampling in handling repetitive genome regions, resulted in insufficient enrichment. Even when *S. mansoni* DNA was retained, genome coverage remained low, indicating that further optimisation would be necessary. However, given the inherent challenges posed by *S. mansoni* genome complexity, it remains uncertain whether adaptive sampling could ever fully replace multiple field washes for effective WGS.

## Data Availability

The raw Nanopore sequencing data (FASTQ files) have been deposited in the NCBI Sequence Read Archive (SRA) and are associated with the following BioSample accessions: SAMN47928699, SAMN47928700, SAMN47928701, SAMN47928702, SAMN47928703, SAMN47928704, SAMN47928705, SAMN47928706, SAMN47928707, SAMN47928708, SAMN47928709, SAMN47928710. These datasets are publicly available and can be accessed through the NCBI SRA linked to BioSample record - PRJNA1250111 – available at: https://www.ncbi.nlm.nih.gov/bioproject/1250111 Zenodo: Table S1. Sequencing summary, taxonomic classification, and coverage metrics for *Schistosoma mansoni* miracidia samples using Nanopore adaptive sampling, https://doi.org/10.5281/zenodo.15290569 ^43^. This dataset contains results from post-sequencing analysis used to evaluate the efficiency of adaptive sampling for enriching *Schistosoma mansoni* DNA and the taxonomic classification of DNA fragments using various bioinformatics tools. It also contains all data used to create the graphs in the manuscript. Data are available under the terms of the Creative Commons Attribution 4.0 International license (CC-BY 4.0).
