# Supplementary material for: How useful is Nanopore adaptive sampling for sequencing Schistosoma mansoni miracidia?
Source: Wellcome Open Res. 2025 May 16;10:239. [Version 1] doi: 10.12688/wellcomeopenres.24094.1 (PMC12149812; doi:10.12688/wellcomeopenres.24094.1)
Supplement: Peer review history

# Reviewer report: Reviewer response for version 1

**DOI:** 10.21956/wellcomeopenres.26581.r123821

**Published:** 2025-06-16

**Authors:** Kaylee S. Herzog

**Affiliations:** 1 University of Nebraska Medical Center, Omaha, Nebraska, USA

**Summary**

 This manuscript represents a valuable contribution to the growing body of literature on the use of next-generation sequencing technologies for population-level studies on consequential helminths. The authors compare two preservation approaches (washed versus unwashed) for field-collected miracidia of *S. mansoni. *Nucleic acid is extracted from FTA card punches representing individual miracidia, amplified via whole genome amplification, and used to generate individual Oxford Nanopore Technologies (ONT) next-generation sequencing libraries. In an effort to increase the proportion of on-target reads in final datasets, these libraries are then sequenced employing ONT’s “adaptive sampling” feature. The authors find that adaptive sampling does not replace the benefit of washing miracidia in the field prior to preservation, as unwashed samples had significantly lower alignment metrics as compared to washed samples. Additionally, the authors describe an interesting and important limitation in the use of adaptive sampling technology with large, complex eukaryotic genomes and low input sample types. My general and specific comments are largely minor, and mainly focus on improving clarity, accuracy, and context.

**General Comments**

- Throughout the manuscript, the authors reference whether adaptive sampling “provides sufficient *S. mansoni* DNA”. Adaptive sampling does not change the amount of nucleic acid in a sample–only the proportion of on-target library fragments sequenced. This is a nuanced clarification, to be sure, but I believe an important one.

- I suggest revising “Nanopore sequencing technologies”, “Nanopore Technologies” and “Nanpore” to the company’s appropriate name “Oxford Nanopore Technologies” throughout
- In the Methods and Results sections on the Kraken2 comparisons, the reader would benefit from more detailed and specific language, as these sections are a bit difficult to interpret as written.

- Throughout, the authors attribute low alignment rates to a lack of miracidium in the sample or a failure of adaptive sampling technology. However, given that whole genome amplification was employed prior to library preparation, it could also be that a miracidium was present in the sample, but whole genome amplification preferentially amplified off-target DNA.

**Specific Comments**

 Abstract

- “larval stage miracidia” is redundant

Introduction

- Second paragraph–I caution against referring to inference from whole genome sequencing as a “more zoomed-in approach”–especially when comparing it against microsatellite panels and amplicon-based assays, as these are definitionally a more “zoomed in” version of whole genome sequencing. Perhaps “a more comprehensive and flexible approach” would be more appropriate here?
- It would be worth mentioning whole genome amplification (WGA) here in the introduction, and its use in generating whole genome sequence data from low-input sample types. This method has previously been applied in schistosomes (e.g., Berger et al. 2021)(refer 1) to great effect, and is importantly leveraged in this study as well.
- “enables selective enrichment of DNA regions or organisms in real-time sequencing” is a bit confusing as written, as organisms are not being enriched. Consider revising to “enables selective enrichment of specified target sequences in real-time”
- In Paragraph 7, it is misleading to say that adaptive sampling is “removing the contamination from samples”. Any contamination is still present in the sample, it is just not sequenced.

Methods

- It would be worth clarifying the process of elution after WGA–if samples were purified following WGA using AmPure Beads (as a downstream sentence suggests) then this sentence should be moved up to immediately follow the WGA section, and thus help the reader understand how a 100µL elution volume was reached.
- Though potentially outside the scope of this study, I wonder if the authors would have had greater sample sizes for comparing across treatment groups if a qPCR assay had been used to screen samples after WGA and prior to library preparation, to ensure libraries were only generated from samples where *S. mansoni  *nucleic acid was demonstrably present.
- In the library preparation and sequencing section, I strongly recommend clarifying the input to library preparation at first mention. It is stated that 1µg of DNA was used as input–if this is this 1µg of DNA per sample, that should be stated explicitly, as should the number of libraries generated.
- I recommend replacing “molecular concentration” with “molarity”

Results

- “below 1.6% *Schistosoma *DNA enriched during adaptive sampling” is difficult to understand as written. Would “less than 1.6% of reads aligning to the *S. mansoni* reference genome” be appropriate?
- I suggest revising the subheader “Kraken2 estimates a higher proportion of *S. mansoni* DNA compared to adaptive sampling” to “A high proportion of reads rejected by adaptive sampling were identified as *Schistosoma* spp. by Kraken2” or similar

Discussion

- Small typo in paragraph 4–revise “as were not economically viable” to “as they were not economically viable”
- It is clear that, overall, samples had low breadth and depth of coverage across the target reference genome. However, given the population-level context in which the authors couch this study in the abstract and introduction, it may be worth some discussion of how much breadth and depth of coverage is really “enough” to call SNPs in *S. mansoni*. For example, how many sites *were* sequenced sufficiently to allow for comparisons across samples, and did this change between the washed and unwashed treatment groups?

Is the work clearly and accurately presented and does it cite the current literature?

Partly

If applicable, is the statistical analysis and its interpretation appropriate?

Yes

Are all the source data underlying the results available to ensure full reproducibility?

Yes

Is the study design appropriate and is the work technically sound?

Yes

Are the conclusions drawn adequately supported by the results?

Yes

Are sufficient details of methods and analysis provided to allow replication by others?

Yes

Reviewer Expertise:

Parasite population genomics; Oxford Nanopore Technologies MinION sequencing

I confirm that I have read this submission and believe that I have an appropriate level of expertise to confirm that it is of an acceptable scientific standard.

## References

[1] : Whole-genome sequencing of Schistosoma mansoni reveals extensive diversity with limited selection despite mass drug administration. *Nature Communications* .2021;12(1) : 10.1038/s41467-021-24958-0 10.1038/s41467-021-24958-0 PMC834651234362894

# Reviewer report: Reviewer response for version 1

**DOI:** 10.21956/wellcomeopenres.26581.r123823

**Published:** 2025-06-09

**Authors:** Hanwen Yang

**Affiliations:** 1 Biology, Saint Louis University College of Arts and Sciences, St. Louis, Missouri, USA

**Peer Review**

 In this article, the authors employ Nanopore adaptive sampling and Kraken2 to analyze sequencing data from 12 *Schistosoma mansoni* miracidia samples—six of which were washed and six unwashed. The study aims to evaluate whether Nanopore adaptive sampling can effectively enrich miracidia DNA and compares its performance with Kraken2 in terms of the proportion of *S. mansoni* reads detected. The experimental design is well-conceived, addressing the real-world challenge of acquiring sufficient *S. mansoni* miracidia DNA from patient and exploring the importance of washing steps in sample preparation.

**Major Points:**

 As demonstrated in Figure 2 and elaborated in the Discussion section, the breadth and depth of coverage of Nanopore adaptive sampling are insufficient for whole genome assembly. The authors attribute this limitation to the constraints of adaptive sampling and the ow cell number in miracidium, which yields limited DNA. This is a valid explanation—assembling a complete genome from such a small amount of starting material is highly challenging, especially when considering the additional complication of environmental contamination.

 The authors also discuss potential genomic differences between laboratory-cultured *S. mansoni* and field-isolated samples used in this study, further justifying the relevance of their experimental approach and highlighting the study's translational value.

 However, the paper lacks downstream analysis of the generated data. Since the genome assembly from individual samples is not feasible due to limited DNA, and given that all miracidia samples originate from a single patient (as mentioned in the Methods), it would be logical to consider integrating or pooling the data across samples for aggregate analysis. This approach could potentially improve genome coverage and provide additional insights.

Is the work clearly and accurately presented and does it cite the current literature?

Yes

If applicable, is the statistical analysis and its interpretation appropriate?

Partly

Are all the source data underlying the results available to ensure full reproducibility?

Yes

Is the study design appropriate and is the work technically sound?

Yes

Are the conclusions drawn adequately supported by the results?

Yes

Are sufficient details of methods and analysis provided to allow replication by others?

Yes

Reviewer Expertise:

plant science, C. elegans, epigenetics.

I confirm that I have read this submission and believe that I have an appropriate level of expertise to confirm that it is of an acceptable scientific standard.

# Reviewer report: Reviewer response for version 1

**DOI:** 10.21956/wellcomeopenres.26581.r123825

**Published:** 2025-06-02

**Authors:** Lucas Huggins

**Affiliations:** 1 The University of Melbourne, Parville, Australia

Comments to the Authors

 This manuscript reports on the feasibility of using Oxford Nanopore Technologies (ONT) MinION Mk1B sequencing device with adaptive sampling to be able to obtain whole S. mansoni genomes from miracidia. The authors report on the efficacy of whole genome sequencing with and without the use of wash steps to remove debris from single miracidia to thereby reduce the chance of off-target amplification of contaminant DNA, e.g. from bacteria. Moreover, the authors detail some interesting methodological findings and comparisons, for example that the Kraken classifier was able to detect more S. mansoni reads than ONT’s adaptive sampling. Overall, the manuscript is excellently written, clear, informative and contextually well-grounded for the field. This research provides the scientific community with a useful resource with which to guide the development of experiments for whole genome sequencing of different parasite life cycle stages that have low quantities of starting DNA. I have some minor comments and points that could be clarified, as well as some suggestions/ideas for further experimentation that I hope the authors will find useful. 

 Minor Amendments

 The version of the manuscript provided to me for review did not have line numbers, so I have tried to refer to the correct paragraph to which my comments pertain. 

 -    Introduction – overall the Intro is well-written and sets the context of the paper well. However, I found it overly long and with unnecessary detail. In particular, Intro paragraph 2, 3 and 6 could be significantly shortened, especially the explanation of how ONT adaptive sampling works (given that this technology has been available since ~2019).

 -    Intro para 1 – Schistosoma is also responsible for considerable mortality (not just morbidity).   

 -    Intro para 6 – “Nanopore Technologies” is not the name of the company. It should be corrected do Oxford Nanopore Technologies throughout (or an appropriate acronym). 

 -    Aims of the project are clearly articulated at the end of the Introduction. 

 -    Methods “whole genome amplification” - the authors refer to the WGA kit used. WGA kits such as this can generate substantial branching heteroduplexes due to the strand displacement activity of the polymerase. The exact ONT protocol used to process this WGA DNA is not referred to – was a protocol used that includes a T7 endonuclease step to reduce some of this branching? Without the T7 step pores will become clogged more rapidly during sequencing (and therefore result in less data) which may explain the result mentioned by the authors in the second paragraph of the “library prep and sequencing” section. A link or reference to the exact ONT protocol used would be a good addition. Additionally, was a DNA shearing step considered/explored at any stage? This could also improve final WGA DNA yield. 

 -    Methods “post-sequencing analysis" – paragraph 3. The authors report the construction of a custom BLAST database with Schistosoma DNA only. This was used to further investigate some of the Kraken2 identified Schistosoma sequences. Given that BLASTn analysis always returns a top hit result, irrespective of final scores, it would be important to include sequences from contaminant organisms such as bacteria, protozoa, etc. in this bespoke BLAST database, so as to prevent spurious classification of non-Schistosoma sequences with BLASTn. This links to a later comment of mine questioning how BLAST classifications were considered and if identity/query cover thresholds were determined in making final classifications. 

 -    Results – a table showing the amount of data generated per miracidia sample tested should be added. This would include total raw data generated, total Gb, total ‘passed’ reads (and the Q-score used to determine a passed read), sequencing time, etc. Lots of valuable run performance data can be obtained from the ONT sequencing reports generated that would be valuable for readers to assess run performance. 

 -    Results paragraph 1 – could a S. mansoni specific qPCR not be used to assess whether individually extracted miracidia have S. mansoni DNA prior to WGA? If a qPCR targeting a highly repetitive region were used this could clarify the authors’ concerns about some samples having no S. mansoni DNA (or potentially an adaptive sampling failure) and provide better support for the categorisation detailed in paragraph 1. This would save further work and could also be used to assess the performance of different DNA extraction procedures. 

 -    Results paragraph 3 – more detail should be provided on how BLAST analysis was used to accurately classify kraken2 identified S. mansoni sequences that were missed by adaptive sampling. For example, what % identity and across what sequence length, i.e. query cover, was deemed sufficient to confidently classify these sequences using BLAST against the authors bespoke database? This links to my prior Methods query, as the absence of contaminant species’ DNA in the bespoke database may mean that some non-Schistosoma sequences are classified as Schistosoma. This is because the BLAST database has nothing else with which to compare them to and will always return a set of top hits/classifications. Such points should be explored and clarified in the text. This comment is also pertinent to the BLAST key in figure 1C. How were these classifications determined, i.e. at what threshold? Was DNA filtered if it was below a certain length, i.e. too short or non-specific/repetitive, before it was classified? Short and non-specific regions being classified by BLAST against a Schistosoma-only database could have led to some reads being classified as other Schistosoma species (i.e. not S. mansoni) as some genomic regions will be highly conserved across this genus. This point also links to paragraph 3 of the Discussion.  

 -    Results paragraph 4 – whether certain contaminant species, e.g. bacteria, occurred repeatedly and in excess should be mentioned. For example, some DNA extraction kits and amplification reagents can be manufactured with large amounts of endogenous bacterial contamination, see the concept of the “kitome”:

 https://doi.org/10.1186/s40168-017-0267-5

 https://doi.org/10.1186/s12866-020-01839-y

 If inappropriate reagents/kits are used then these can seriously affect the ability to retrieve NGS data of relevance from low starting input DNA samples, such as those in the authors’ research. Appropriate use of controls, e.g. kit/reagent only negative controls should be discussed as these may impact the final outcome of the experiment. Have the authors trialled different kits/approaches with this in mind? 

 -    Results (final 2 paragraphs) – It is interesting that average WGS coverage is so similar between the washed and unwashed samples even though substantially more reads were accrued by adaptive sampling when miracidia were washed. The benefits in terms of amount of data gained when samples were washed does not seem translate to better WGS coverage.

 -    Results – Table 2 – asterisks highlighting the samples that were excluded or deemed as not having miracidia DNA would be a good addition. 

 -    Results – Fig 2, whilst not the fault/domain of the authors figure 2 was too small for me to assess in the review copy I was provided with. This image is very data rich and should be larger in the final version of this manuscript. 

 -    Discussion (general ideas/comments) – could nanopore adaptive sampling parameters be loosened/modified to try and increase the number of S. mansoni sequences that are detected by adaptive sampling? and therefore minimise the discrepancy between this approach and the reads identified by kraken2. Was this attempted? Additionally, could the inverse approach be attempted, i.e. host/contaminant depletion? – some authors have found that this works better than enrichment and could be achieved if common bacterial contaminants were identified. Additional, enrichment-depletion approaches could be used in tandem, for example the use of Cas9 enzymes to remove abundant contaminant sequences or further exploration of different DNA extraction kits that may be better suited to WGA from small DNA starting quantities. 

 From experience in our laboratory where we conduct WGA from single nematode eggs, we have found that preservation on FTA cards actually leads to reductions in extracted DNA. We have done comparisons with single hookworm egg extractions using eggs preserved on FTA cards and those extracted directly from PBS using the QIAamp DNA Micro Kit, with both eluted in ~30ul of buffer. We found FTA preservation greatly reduced the quantity of DNA extracted from single eggs. Hookworm qPCRs used to compare extraction success returned Ct values of ~23-24 from non-FTA preserved samples versus Ct values of 35-36 for FTA preserved samples. Given hookworm eggs comprise ~6-8 cells I would expect that such methods would be valuable within the authors’ research context.

Is the work clearly and accurately presented and does it cite the current literature?

Yes

If applicable, is the statistical analysis and its interpretation appropriate?

Yes

Are all the source data underlying the results available to ensure full reproducibility?

Yes

Is the study design appropriate and is the work technically sound?

Yes

Are the conclusions drawn adequately supported by the results?

Yes

Are sufficient details of methods and analysis provided to allow replication by others?

Yes

Reviewer Expertise:

I am an expert in NGS-based diagnostics for parasite detection

I confirm that I have read this submission and believe that I have an appropriate level of expertise to confirm that it is of an acceptable scientific standard.
